# Effect of structural stability on endolysosomal degradation and T‐cell reactivity of major shrimp allergen tropomyosin

**DOI:** 10.1111/all.14410

**Published:** 2020-06-18

**Authors:** Sandip D. Kamath, Sandra Scheiblhofer, Christopher M. Johnson, Yoan Machado, Thomas McLean, Aya C. Taki, Paul A. Ramsland, Swati Iyer, Isabella Joubert, Heidi Hofer, Michael Wallner, Josef Thalhamer, Jennifer Rolland, Robyn O’Hehir, Peter Briza, Fatima Ferreira, Richard Weiss, Andreas L. Lopata

**Affiliations:** ^1^ Australian Institute of Tropical Health and Medicine James Cook University Townsville Qld Australia; ^2^ Department of Biosciences University of Salzburg Salzburg Austria; ^3^ MRC Laboratory of Molecular Biology Cambridge UK; ^4^ Centre of Blood Research University of British Columbia Vancouver BC Canada; ^5^ School of Science RMIT University Melbourne Vic. Australia; ^6^ Faculty of Veterinary and Agricultural Sciences University of Melbourne Melbourne Vic. Australia; ^7^ Department of Physiology University of Melbourne Melbourne Vic. Australia; ^8^ Department of Immunology and Pathology Central Clinical School Monash University Melbourne Vic. Australia; ^9^ Department of Allergy, Immunology and Respiratory Medicine Central Clinical School Monash University Melbourne Vic. Australia; ^10^ Alfred Hospital Melbourne Vic. Australia

**Keywords:** cross‐reactivity, endolysosomal degradation, shrimp allergy, T cell, tropomyosin

## Abstract

**Background:**

Tropomyosins are highly conserved proteins, an attribute that forms the molecular basis for their IgE antibody cross‐reactivity. Despite sequence similarities, their allergenicity varies greatly between ingested and inhaled invertebrate sources. In this study, we investigated the relationship between the structural stability of different tropomyosins, their endolysosomal degradation patterns, and T‐cell reactivity.

**Methods:**

We investigated the differences between four tropomyosins—the major shrimp allergen Pen m 1 and the minor allergens Der p 10 (dust mite), Bla g 7 (cockroach), and Ani s 3 (fish parasite)—in terms of IgE binding, structural stability, endolysosomal degradation and subsequent peptide generation, and T‐cell cross‐reactivity in a BALB/c murine model.

**Results:**

Tropomyosins displayed different melting temperatures, which did not correlate with amino acid sequence similarities. Endolysosomal degradation experiments demonstrated differential proteolytic digestion, as a function of thermal stability, generating different peptide repertoires. Pen m 1 (T_m_ 42°C) and Der p 10 (T_m_ 44°C) elicited similar patterns of endolysosomal degradation, but not Bla g 7 (T_m_ 63°C) or Ani s 3 (T_m_ 33°C). Pen m 1–specific T‐cell clones, with specificity for regions highly conserved in all four tropomyosins, proliferated weakly to Der p 10, but did not proliferate to Bla g 7 and Ani s 3, indicating lack of T‐cell epitope cross‐reactivity.

**Conclusions:**

Tropomyosin T‐cell cross‐reactivity, unlike IgE cross‐reactivity, is dependent on structural stability rather than amino acid sequence similarity. These findings contribute to our understanding of cross‐sensitization among different invertebrates and design of suitable T‐cell peptide‐based immunotherapies for shrimp and related allergies.

## Graphical abstract

Allergenic tropomyosins have dissimilar structural stabilities despite having high amino acid sequence similarity. Tropomyosins are differentially degraded in the endolysosomal compartment of antigen‐presenting cells due to differences in their structural stability. Tropomyosin T‐cell cross‐reactivity, unlike IgE antibody cross‐reactivity, is dependent on structural stability rather than amino acid sequence similarity.

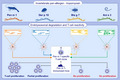

## 1 INTRODUCTION

The tropomyosin protein family is one of the largest allergen families containing over 60 identified and characterized allergens.^1^ Tropomyosin exhibits a high degree of structural conservation between species.^2^ Several studies have shown clinical cross‐reactivity between crustaceans, mollusks, insects, mites, and nematodes is due mainly to shared IgE (B‐cell) epitopes of tropomyosin.^3^, ^4^, ^5^ However, there is a lack of understanding whether this high degree of structural and sequence conservation among tropomyosins would also lead to cross‐reactive T‐cell epitopes. Currently, T‐cell epitopes have only been elucidated for shrimp tropomyosin with little or no data for other allergenic sources, making analysis of T‐cell cross‐reactivity challenging.^6^, ^7^, ^8^

In the tropomyosin family, crustacean and mollusk shellfish tropomyosins are major allergens, particularly shrimp tropomyosin (Pen m 1), with more than 80% of shrimp‐allergic patients being sensitized to this allergen. These major food allergens have been shown to be extremely thermostable and to withstand food‐processing activities.^9^, ^10^ Some other invertebrate tropomyosins from sources such as mites and insects are considered only as minor allergens.

Tropomyosins are alpha‐helical coiled‐coil proteins that generally exist as stable dimers. Structural stability of allergenic proteins has been shown to have a direct impact on their allergenicity through differential endolysosomal degradation and subsequent generation of allergen‐derived peptides for MHC Class II presentation.^11^ It remains unclear, whether the different structural properties of the various closely related tropomyosins also would affect their T‐cell cross‐reactivity.

In this study, four allergenic tropomyosins, from shrimp (Pen m 1), house dust mite (Der p 10), cockroach (Bla g 7), and Anisakis (food borne parasite) (Ani s 3) were investigated for their thermal and proteolytic stability, and their processing into peptides was analyzed *in vitro* and *in vivo*. T‐cell cross‐reactivity of Pen m 1–derived peptides to other tropomyosins was assessed in a murine model. The tropomyosins, although heat‐stable, showed different melting temperatures that were pH‐dependent. Exposure to endolysosomal proteases demonstrated that tropomyosins with similar thermal stabilities also showed a similar speed and pattern of degradation. In a murine model using Pen m 1 peptide‐specific T‐cell clones with conserved sequence identity, we found only limited T‐cell cross‐reactivity with Der p 10, and none with Bla g 7 and Ani s 3. Highly conserved invertebrate tropomyosins may share IgE epitopes leading to clinical cross‐reactivity; however, presence of shared identical T‐cell epitopes seems to be dependent on similarities in structural stability as opposed to amino acid sequence identity. Our findings have implications for understanding possible modes of sensitization as well as the design of suitable tropomyosin preparations for specific immunotherapy for shrimp and related allergies.

## 2 MATERIALS AND METHODS

### 2.1 Cloning, expression, and purification of tropomyosins

The amino acid sequences from the open reading frames for tropomyosin were used for black tiger shrimp (*Penaeus monodon*) accession number ADM34184, house dust mite (*Dermatophagoides pteronyssinus*) accession number ACI32128, German cockroach (*Blattella germanica*) accession number AAF72534.1, and Anisakis parasite (*Anisakis simplex*) accession number Q9NAS5. Detailed methods for recombinant protein expression and purification are outlined in Appendix S1.

### 2.2 Patient recruitment and IgE binding analysis

To analyze IgE binding to the different tropomyosins, 17 shellfish‐allergic patients were recruited at The Alfred Hospital Allergy Clinic, Melbourne, Victoria, Australia, with a positive serum shrimp‐specific IgE (≥0.35 kU/L; ImmunoCAP^®^, Phadia). Serum from a nonatopic donor recruited at the Translational Research Facility, James Cook University, was used as a negative control (Table S1). Written informed consent was obtained from all participants, and patient anonymity was preserved. Ethics approval was obtained from the Ethics Committees of James Cook University (Project numbers H4313 and H6829), The Alfred Hospital (Project number 192/07), and Monash University (MUHREC CF08/0225). IgE recognition of the different tropomyosins was investigated using grid immunoblotting^12^ as described in Appendix S1.

### 2.3 Biophysical characterization of allergenic tropomyosins

The alpha‐helical structure and thermal denaturation of tropomyosins were compared and analyzed using circular dichroism (CD) spectroscopy. The effects of different pH conditions on protein melting temperatures was analyzed using differential scanning calorimetry (DSC) and differential scanning fluorimetry (DSF). Protein molecular mass under different pH conditions was determined using size exclusion chromatography coupled to multi‐angle light scattering (SEC‐MALS). The detailed methodology for biophysical characterization of tropomyosins is given in the Appendix S1.

### 2.4 Endolysosomal degradation of tropomyosins

Endolysosomal degradation assays were performed as described previously.^13^ Briefly, 5 µg of purified protein was mixed with 8 µg of isolated microsomal fraction from the JAWSII cell line in 50 mmol/L citrate buffer (pH 5.2 or pH 4.5) and 2 mmol/L dithiothreitol. Bet v 1 was used as a nontropomyosin control allergen, and to confirm *in vitro* degradation assay condition reproducibility. Degradation was monitored over time using SDS‐PAGE and Coomassie staining. The pool of peptides generated in the degradation assay was assessed by mass spectrometry using a Q‐Exactive Orbitrap Mass Spectrometer (Thermo Fisher Scientific) and nano‐HPLC (Dionex Ultimate 3000, Thermo Fisher Scientific). Detailed methods are provided in Appendix S1. To visualize the digestion‐generated peptide data, the peptide sequences were mapped against the full‐length amino acid sequences of the respective tropomyosins using MS tools.^14^ The speed and intensity of peptide generation were visualized using plot.ly.

### 2.5 Generation of Pen m 1 overlapping peptide library and mapping of murine T‐cell epitopes of Pen m 1

To map the T‐cell epitopes of Pen m 1, an overlapping peptide library was generated with 15‐mer peptides with an offset of three amino acids spanning the entire length of Pen m 1 (Mimotopes). BALB/c mice were immunized with whole Pen m 1, and the splenocyte proliferation assay was performed using the overlapping peptide library. T‐cell reactive regions were mapped, based on CFSE‐based T‐cell proliferation and IL‐2 release as correlates. The detailed methodology is provided in Appendix S1.

### 2.6 Generation of Pen m 1 conserved region‐specific T‐cell hybridomas

Based on T‐cell proliferation data for the Pen m 1 peptide library, two peptide sequences were chosen for the generation of Pen m 1–specific T‐cell hybridomas. These regions (peptides 67 ^199‐^VVGNNLKSLEVSEEK^‐213^ and 82 ^244‐^RSVQKLQKEVDRLED^‐258^) were chosen based on (a) positive T‐cell proliferation to these peptides and (b) high amino acid sequence similarity to corresponding regions in Der p 10, Bla g 7, and Ani s 3 (Figure S1). The Pen m 1–specific hybridomas were used as a tool to investigate whether the different tropomyosins share cross‐reactive T‐cell epitopes at these regions. The detailed methodology for peptide immunization and generation of hybridomas is provided in Appendix S1.

### 2.7 Processing and T‐cell reactivity of tropomyosin homologues using Pen m 1–specific T‐cell hybridomas

To investigate whether dendritic cell processing of the different tropomyosins results in the presentation of Pen m 1–cross‐reactive T‐cell epitopes, co‐cultures of Pen m 1–specific T‐cell hybridoma clones with GM‐CSF grown bone marrow dendritic cells (BMDCs) and serial dilutions of the four tropomyosins were set up in 96‐well U‐bottom plates. 10^5^ T cells per well from clones 67‐1.A2 and 82‐3.C5 were incubated overnight with 2 × 10^4^ GM‐CSF BMDCs and concentrations of 50, 10, or 2 µg/mL of Pen m 1, Der p 10, Ani s 3, and Bla g 7 respectively, in triplicate. Control wells received either medium alone, peptides 67 and 82 at 10 µg/mL, or an equal volume of peptide diluent (DMSO). Culture supernatants thereof were removed and analyzed for IL‐2 production as a correlate for T‐cell activation using an ELISA MAX mouse IL‐2 set (BioLegend).

## 3 RESULTS

### 3.1 Allergenic tropomyosins are highly conserved proteins with frequent IgE co‐sensitization in shrimp‐allergic patients

**Figure 1:**
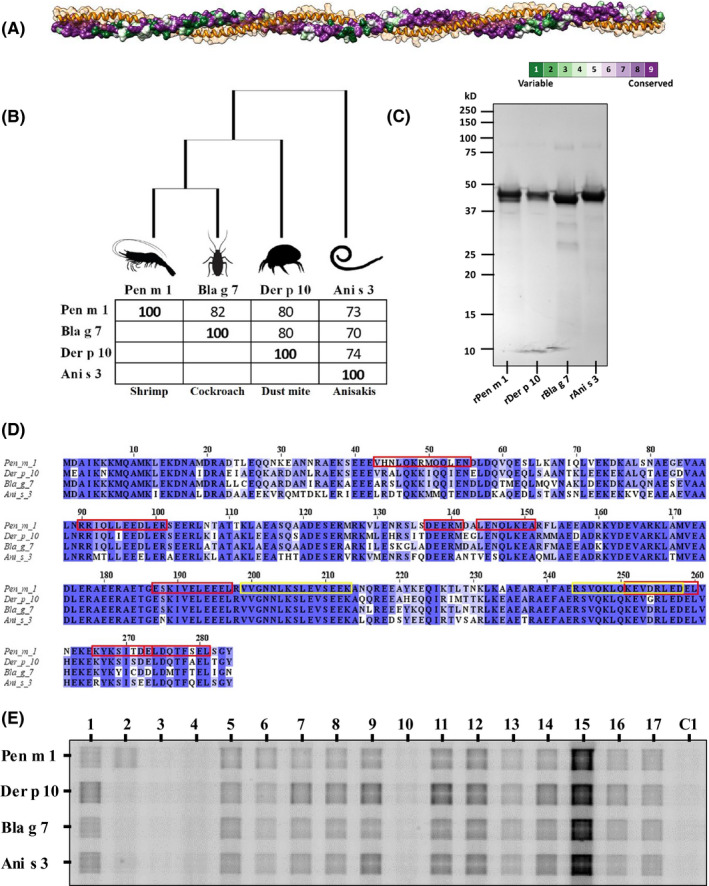
Invertebrate tropomyosins investigated in this study. A, A homology model of tropomyosin displaying the alpha‐helical coiled‐coil structure using a ribbon/space‐fill model (orange) and the patterns of sequence conservation shown using ConSurf model. B, A phylogenetic tree and percent identity grid for invertebrate tropomyosins investigated in this study. C, SDS‐PAGE Coomassie‐stained gel profile of purified tropomyosins. D, Multiple sequence alignment using Clustal Omega algorithm of Pen m 1, Der p 10, Bla g 7, and Ani s 3 showing conserved amino acid residues. Pen m 1 IgE‐binding epitopes are denoted by red boxes.^15^ Yellow boxes indicate Pen m 1 peptides 67 and 82 selected for T‐cell cross‐reactivity experiments. E, IgE grid immunoblotting using serum from shrimp‐allergic patients (1‐17) and one healthy donor (C1) to demonstrate presence or absence of IgE co‐sensitization to invertebrate tropomyosins

The tropomyosins selected for this study have a high degree of conservation with 70% or more amino acid sequence identity (Figure 1A,B). These allergens were expressed in a bacterial expression system and purified as recombinant proteins using affinity and size exclusion chromatography (Figure 1C). A multiple sequence alignment of the four tropomyosins revealed the high degree of conservation in the IgE epitopes of shrimp tropomyosin as characterized previously (Figure 1D).^15^ Five out of eight IgE epitopes from Pen m 1 were highly conserved in Der p 10, Ani s 3, and Bla g 7. To evaluate IgE antibody co‐sensitization to the different tropomyosins, IgE grid immunoblotting was performed using sera from shrimp‐allergic patients (Figure 1E). Thirteen out of 17 subjects demonstrated IgE binding to all tropomyosins. Only one subject showed mono‐sensitivity to Pen m 1. Interestingly, 8/17 subjects had stronger IgE binding to Der p 10 as compared to Pen m 1 based on densitometric analysis.

### 3.2 Tropomyosins have differential structural stability and pH‐dependent aggregation

**Figure 2:**
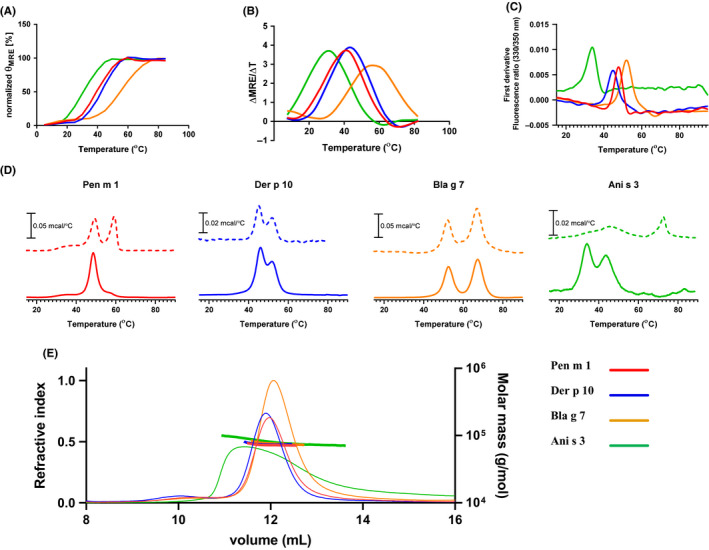
Structural characterization of tropomyosins. Analysis of thermal stability of Pen m 1, Der p 10, Bla g 7, and Ani s 3 using (A) CD spectroscopy, observing changes in MRE at 222 nm, and (B) first derivative of MRE from 15 to 85°C. Analysis of thermal stability by using (C) DSF and (D) observing changes in heat capacity using (DSC) at pH 5.2 (dotted line) and pH 7.4 (solid line). E, Analysis of tropomyosins by SEC‐MALS indicated molar masses consistent with essentially full occupancy of dimer at pH 7.4. The RI chromatograms on the left axis (thin lines) while the evaluated molar masses shown with the right axis (thick horizontal lines)

Using CD spectroscopy, mean residual ellipticity (MRE) at 222 nm was monitored. Pen m 1 and Der p 10 had similar melting temperatures (inflection points) of 42°C and 44°C, respectively. Although Bla g 7 showed a higher melting temperature (63°C), loss of alpha‐helical structure was initially observed from as early as 40°C (Figure 2A,B). Interestingly, Ani s 3 showed the lowest melting temperature of 33°C with nearly complete loss of alpha‐helical structure by 50°C. DSF and DSC analysis of the tropomyosins confirmed similar melting temperatures for Pen m 1 and Der p 10, as well as the low melting temperature for Ani s 3 at neutral pH as compared to other tropomyosins (Figure 2C, 2D).

The effect of two different pH conditions on melting temperatures was analyzed by DSC (Figure 2D). The specific heat capacity (Cp) change during protein denaturation (mcal/°C) was measured from 10 to 85°C for the invertebrate tropomyosins at pH 5.2 and pH 7.4 in independent experiments. Pen m 1 showed a slight 2°C increase in melting temperature and an additional transition at 60°C at pH 5.2 as compared to pH 7.4. Der p 10 and Bla g 7 did not show any shifts in melting temperatures when analyzed under acidic compared with neutral conditions. Interestingly, Ani s 3 showed a large shift to 73°C at pH 5.2 from 33°C at pH 7.4.

Aggregation states of tropomyosins under acidic (pH 5.2) and neutral (pH 7.4) conditions were assessed using SEC‐MALS. At neutral pH, all four tropomyosins existed as dimers as indicated by the molecular weight calculated from the peaks during size exclusion chromatography (Figure 2E). Ani s 3 showed a broader peak as compared to other tropomyosins. SEC‐MALS was performed as a constant temperature of 25°C, and from melting temperature analysis, it was evident that Ani s 3 would have already started unfolding at this temperature, and thus, we are seeing a complex equilibrium of folded and unfolded protein. At pH 5.2 however, the protein peaks were absent indicating that the tropomyosins were highly charged and/or in an aggregated state, which resulted in their retention in the prefilter or strong binding to the column matrix (data not shown).

### 3.3 Allergenic tropomyosins demonstrate differential pH‐dependent protease‐mediated degradation

**Figure 3:**
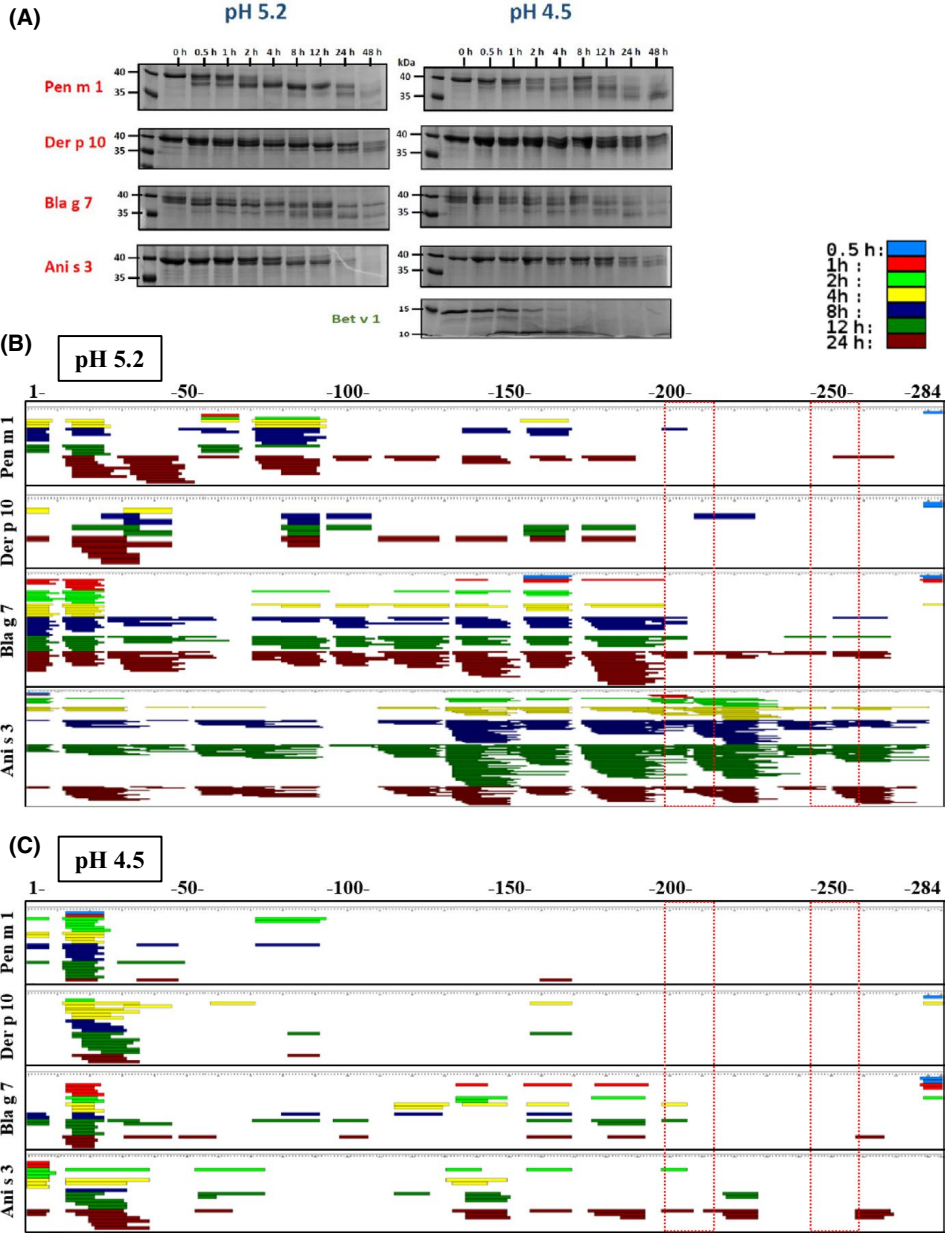
Endolysosomal degradation of tropomyosins using the microsomal fraction of murine dendritic cells (JAWS II) to mimic degradation in antigen‐presenting cells. Coomassie‐stained SDS‐PAGE gel of time‐dependent digestion products of tropomyosins over 48 h; lane 1 indicates molecular ladder (A). Mass spectrometric sequencing of endosomal digestion‐generated tropomyosin peptides mapped against the whole amino acid sequence (numbered 1‐284) for tropomyosins under acidic condition at pH 5.2 (B) and pH 4.5 (C). Peptides generated at various time‐points are depicted in various colors (see color key). Regions corresponding to the immunoreactive Pen m 1 peptides; 67 and 82 are highlighted (dashed boxes)

The endolysosomal degradation assay was performed to compare the peptide profiles generated from tropomyosins in the endolysosomal compartment of antigen‐presenting cells. The four tropomyosins were incubated with the microsomal fraction from JAWSII dendritic cells under different pH conditions (pH 5.2 and pH 4.5) (Figure 3).

At pH 5.2, progressive digestion of native‐sized Pen m 1, Der p 10, and Bla g 7 was observed over 8 hours using SDS‐PAGE. A stable 35‐37 kDa fragment persisted up to 48 hours, while the corresponding fragment of Ani s 3 was completely degraded within 48 hours. At pH 4.5, the rate of digestion of tropomyosins was slower. Der p 10 in particular was stable to enzymatic digestion at this lower pH, with a native‐sized protein persisting even after 48 hours (Figure 3A). Interestingly, the degradation patterns over time suggested progressive degradation from the N‐ and/or C‐terminal ends of the coiled‐coil proteins. In most cases, a residual fragment >35 kDa in size was observed. Tropomyosins in general were more resistant to endolysosomal degradation, particularly Pen m 1, as compared to the nontropomyosin control, Bet v 1 (Figure 3A).

**Figure 4:**
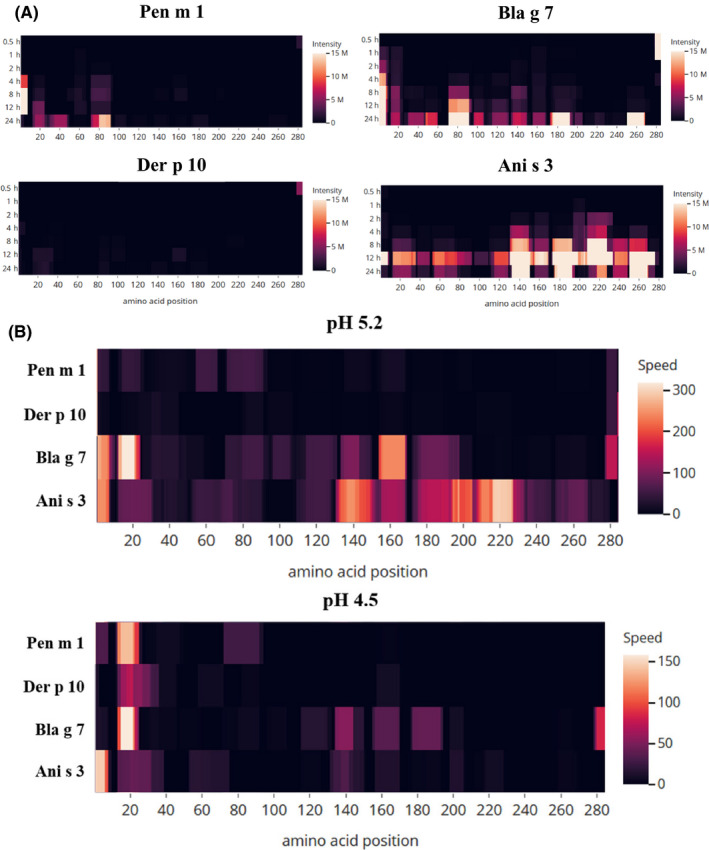
Peptide heat map depicting the intensity at pH 5.2 (A) and speed (B) of generation of tropomyosin‐derived peptides during the endolysosomal degradation assay under acidic conditions. The heat map indicates the position and abundance of the generated peptides as sequenced by mass spectrometric analysis, mapped against the full‐length amino acid sequence of the different tropomyosins

Mass spectrometry‐based peptide sequencing was used to further assess the pattern of peptide generation during degradation at pH 5.2 and pH 4.5 (Figure 3B,C). At pH 5.2, a higher abundance of digestion‐derived peptides was observed than at pH 4.5 for all tropomyosins. For Bla g 7 and Ani s 3, a marked increase in peptide generation was observed after 4 hours at pH 5.2. Pen m 1 and Der p 10 were more slowly degraded compared to Bla g 7 and Ani s 3 and displayed a similar peptide repertoire. Peptides were generated in the early time‐points at the N‐terminal side for all four tropomyosins as predicted using SDS‐PAGE analysis, although the pattern of peptide generation was different at longer time‐points particularly for Bla g 7 and Ani s 3. The speed and intensity of peptide generation correlated with structural stability of the tropomyosins at both pH 5.2 (Figure 4) and pH 4.5 (Figure S2). Ani s 3 and Bla g 7 showed faster uncoiling of the alpha‐helical structure during temperature‐dependent denaturation at lower pH as shown by DSC and CD analysis, correlating with more rapid peptide generation than for the other tropomyosins.

### 3.4 The dominant T‐cell epitope of Pen m 1 shows weak cross‐reactivity with Der p 10 and none with other invertebrate tropomyosins

**Figure 5:**
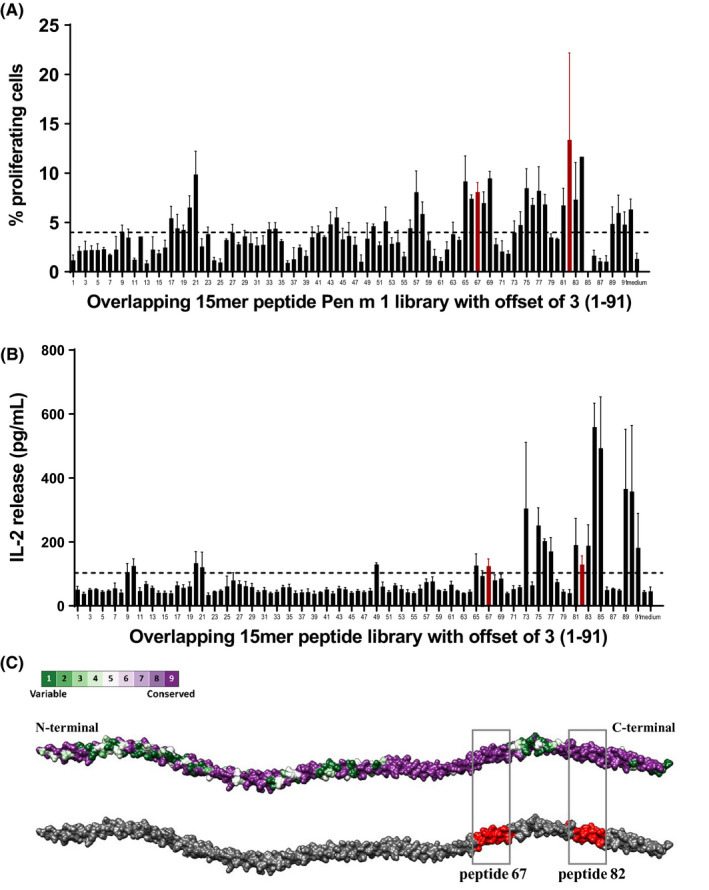
Murine T‐cell epitope mapping of Pen m 1. Immunoreactive regions of Pen m 1 were mapped using overlapping 15‐mer peptides with an offset of 3 amino acids. A, Proliferating T‐cells were analyzed using CFSE dye‐dilution method. B, IL‐2 release in the supernatant was measured using ELISA. Data are shown as mean with standard error of mean (SEM) for three replicate cultures. The cutoff of three standard deviations above mean reactivity of medium‐only control wells is indicated by the dotted line (A, 3.98% proliferating cells or B, 103.2 pg/mL IL‐2). C, A model of tropomyosin representing the amino acid sequence conservation between Pen m 1, Der p 10, Bla g 7, and Ani s 3 generated using ConSurf conservation model in Chimera. The red shaded regions indicate the two T‐cell reactive regions of Pen m 1; peptide 67 and 82 selected for this study that are also highly conserved among the four allergenic tropomyosins

**Figure 6:**
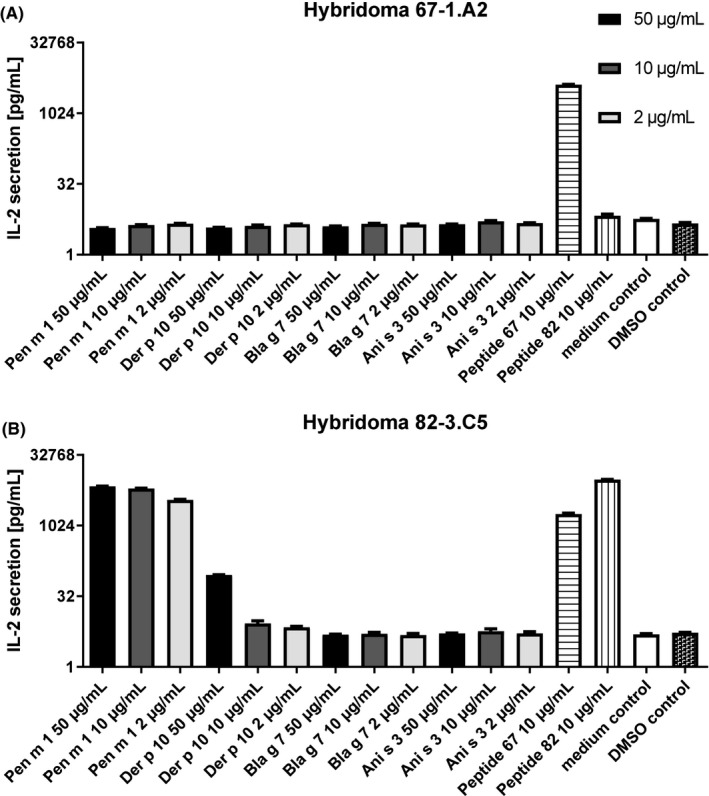
Murine T‐cell cross‐reactivity to allergenic tropomyosins. T‐cell cross‐reactivity of Pen m 1, Der p 10, Bla g 7, and Ani s 3 to T‐cell hybridoma clones specific for peptide 67 and 82 was analyzed by measurement of IL‐2 production upon exposure to protein, peptide controls or medium. Data are shown as mean with standard error of mean (SEM) for three replicate cultures

T‐cell reactive regions of Pen m 1 were first mapped by assessing proliferation and IL‐2 release (Figure 5A,B) from Pen m 1–immunized mouse splenocytes cultured with an overlapping Pen m 1 peptide library. T‐cell reactivity was generally greater to C‐terminal Pen m 1 peptides. Two T‐cell reactive Pen m 1 peptides with identical or near identical sequences for corresponding regions in Der p 10, Bla g 7, and Ani s 3 (67, aa 199‐213, and 82, aa 244‐258) were selected for T‐cell hybridoma generation (Figure S1). To assess whether the invertebrate tropomyosins exhibited T‐cell cross‐reactivity at these conserved regions, the specific T‐cell hybridomas were cultured with all four tropomyosins in separate experiments in the presence of mouse GM‐CSF BMDCs as antigen‐presenting cells, and T‐cell stimulation was assessed by IL‐2 release (Figure 6).

Although peptide 67 (positive control) induced strong stimulation of clone 67‐1.A2, none of the four tropomyosins or peptide 82 did. The clone 82‐3.C5 (generated against peptide 82) showed reactivity to both peptide 67 and peptide 82. This clone showed a response to Pen m 1 at all three tested concentrations, while Der p 10 elicited a response only at the highest concentration tested and Bla g 7 and Ani s 3 did not induce any T‐cell stimulation. Notably, these regions are highly similar (peptide 67:100% for all three homologues; peptide 82:93% for Der p 10, and 100% for Bla g 7 and Ani s 3) among all four tested tropomyosins, and yet cross‐reactivity was not observed. Interestingly, these immunoreactive regions did not concur with the in vitro degradation assay outcomes of all tested tropomyosins where full‐length peptides 67 and 82 were not detected (Figure 3 B,C).

## 4 DISCUSSION

Tropomyosin is a highly conserved structural protein that has been the central focus of immunodiagnostic and therapeutic developments for shrimp allergy. Tropomyosin is the primary sensitizer in various crustacean and mollusk species, and a key player in our understanding of clinical cross‐reactivity in shrimp‐allergic patients on exposure to other invertebrate sources such as insects,^16^, ^17^ mites,^18^ nematodes,^19^ and more recently, even vertebrates such as fish.^20^, ^21^ However, it remains to be elucidated whether other invertebrate tropomyosins can also cross‐react on a T‐cell level.

In this study, we sought to understand the fundamental relationship between allergenic invertebrate tropomyosins in terms of their structural stabilities, endolysosomal degradation patterns, and T‐cell reactivity. Using an animal model, we investigated whether Pen m 1–specific T cells could cross‐react with other invertebrate tropomyosins due to conserved T‐cell epitope regions.

Four tropomyosins were investigated in this study: Pen m 1 (shrimp), Der p 10 (house dust mite), Bla g 7 (cockroach), and Ani s 3 (Anisakis). Through our detailed structural analysis, we demonstrate here that these tropomyosins, despite their conserved amino acid sequences, exhibit different stabilities as reflected in their melting temperatures, while all are known to withstand heat‐processing and retain IgE‐binding capacity. Pen m 1 and Der p 10 showed similar thermal stabilities, whereas Ani s 3 displayed a remarkably low melting temperature, which may be a physiological property of a fish parasite protein that is adapted to a cooler environment. Although Bla g 7 was thermodynamically more stable compared to Pen m 1, it already showed partial unfolding of alpha‐helical structures at 40°C. In contrast to Der p 10 and Bla g 7, an increase in melting temperature was observed for Pen m 1 and Ani s 3 under acidic pH conditions. This variation in stability at acidic pH indicates that some tropomyosins might be degraded differentially during antigen presentation when subjected to increasingly acidic conditions in the early to late endolysosomal compartments after uptake by antigen‐presenting cells.^11^, ^22^ Due to tropomyosins’ propensity to aggregate under pH conditions close to its isoelectric point, analysis at pH 4.5 could not be performed. However, the shift in shrimp tropomyosin’s melting temperature at pH 2 was previously confirmed by James et al.^23^

During allergic sensitization, allergen‐specific peptides, that are generated during the endolyososomal degradative process, are loaded onto MHC class II molecules and displayed on the cell surface for presentation to CD4+ T cells. This eventually results in a Th2 cytokine response and subsequent generation of allergen‐specific IgE. To establish whether the different invertebrate tropomyosins are degraded and processed differently during this stage, an in vitro assay mimicking the endolysosomal degradation was performed.^24^ For the first time, we demonstrate that tropomyosins are degraded differentially and at different speeds depending on pH conditions. The speed and intensity of peptide generation were higher for Ani s 3 and Bla g 7 as compared to Pen m 1 and Der p 10. Ani s 3 in particular showed a low melting temperature, which may have led to enhanced unwinding or fraying of the coiled‐coil structure giving way for more efficient degradation of the peptide bonds. Increased thermodynamic stability did not always translate into increased resistance to in vitro endolysosomal degradation. This was demonstrated by Bla g 7 (closely related to Pen m1 by sequence similarity), which showed highest stability among the tested tropomyosins, but showed lower resistance to proteolytic degradation that was similar to the least stable Ani s 3. Pen m 1 and Der p 10, which showed very similar thermodynamic stability, also displayed similar degradation patterns under both pH conditions. We concluded that similar structural stabilities rather than amino acid sequence similarities resulted in specific endolysosomal degradation patterns and peptide generation.

To further investigate whether differences in biophysical and immunochemical properties of tropomyosins would impact T‐cell reactivity, murine T‐cell cross‐reactivity analysis was performed. Murine T‐cell clones were generated specifically against regions that were T‐cell reactive in Pen m 1 and highly conserved among all four tropomyosins. When BMDCs were cultured with these T‐cell clones together with the four tropomyosins, Bla g 7 and Ani s 3 did not induce any significant response. Der p 10–induced T‐cell stimulation only at the highest tested concentration. Clone 67‐1.A2 did not exhibit any stimulation even on exposure to whole Pen m 1. Surprisingly, peptide 67 was able to induce stimulation in T‐cell clone 82.3.C5 indicating T‐cell receptor (TCR) cross‐reactivity between these two internal regions of Pen m 1. A single TCR has been shown to recognize more than one specific peptide.^25^, ^26^ We conclude that peptide 67 was only identified in the initial screening using whole Pen m 1 due to its cross‐reactivity with the immunodominant region 244‐254, as T‐cell clones specifically raised against peptide 67 fail to be stimulated by all tested tropomyosins. The alpha‐helical coiled‐coil structure imparts features unique to tropomyosins, namely a heptad repeat amino acid sequence.^27^ This feature makes it possible to have regions which have different amino acid sequences but belonging to similar classes that would act as anchor points to the peptide groove of MHC class II molecules. This could result in nonhomologous “nonidentical” cross‐reactive T‐cell epitopes “intra”‐ and “inter”‐tropomyosins. This warrants further investigation. A caveat of this study is that immunoreactive Pen m 1–specific peptides elucidated experimentally by epitope mapping did not concur with the peptides sequenced from the in vitro degradation assay. Peptides in this region were absent for the reactive Pen m1 and Der p 10, and more abundant for the non–cross‐reactive Bla g 7 and Ani s 3. Consequently, T‐cell cross‐reactivity between Pen m 1 and Der p 10 could not be supported by the in vitro degradation outcomes. This suggests that tropomyosin‐generated peptides, fated to be loaded on class II molecules, may be transient and protected by binding to the complex, which would otherwise be degraded rapidly under in vitro conditions.^28^

From a clinical perspective, although avoidance of any shrimp‐based foods can prevent further sensitization and IgE‐mediated reactions in shrimp‐allergic patients, it is not known whether environmental exposure to dust mite or insect‐derived tropomyosin via inhalation, or to the fish parasitic nematode Anisakis tropomyosin via ingestion, could lead to further priming and re‐stimulation of a shrimp‐specific allergic response. T‐cell cross‐reactivity has recently been demonstrated for nut allergy, where cashew‐specific CD4+ T cells from cashew‐allergic subjects can cross‐react with tree nut allergens such as hazelnut and pistachios due to homologous T‐cell epitopes with high amino acid identity.^29^ Similarly, pollen‐related food allergens were shown to induce Bet v 1‐specific T‐cell responses in pollen‐allergic subjects, which might perennially boost IgE levels out of the pollen season due to consumption of such foods.^30^

In summary, invertebrate tropomyosins do not share similar stabilities as a function of amino acid sequence similarity. Protein stability differences may be the prime reason for differential degradation and antigen presentation of various allergenic tropomyosins, leading to generation of nonidentical T‐cell epitopes. Our study concludes that T‐cell cross‐reactivity among tropomyosins may be associated to their structural stability rather than amino acid sequence similarity, which is more the case for IgE cross‐reactivity of allergens.^4^

Shellfish allergy, particularly to shrimps, affects more than 3% of the population and is frequently associated with life‐long sensitivity and severe allergic reactions.^31^, ^32^, ^33^ Exposure to other invertebrate sources such as house dust mites or insect‐based food sources^34^, ^35^ may put this population at risk of developing severe allergies, even upon avoiding shellfish. It is important to understand T‐cell cross‐reactivity among these invertebrate sources and the risk of being cross‐sensitized. This study contributes to our understanding of different biophysical factors that play a role in generation of tropomyosin T‐cell epitopes and may assist in the design of novel T‐cell peptide‐based therapies designed to tackle shrimp allergy and other related allergen sources.

## CONFLICT OF INTEREST

SK and AL report grants from National Health and Medical Research Institute, during the conduct of the study. FF reports nonfinancial support from Priority Program “Allergy‐Cancer‐BioNano Research Centre” of the University of Salzburg, during the conduct of the study; personal fees from HAL Allergy, personal fees from Swiss Institute of Allergy and Asthma Research (SIAF), and personal fees from AllergenOnline, outside the submitted work. RW reports grants from Austrian Science Fund (FWF), during the conduct of the study. All other co‐authors have nothing to disclose.

## AUTHORS CONTRIBUTION

SK, FF, RW, JT, and AL conceptualized and designed the research objectives. SK, SI, AT, and TM expressed and purified the recombinant allergens. CMJ conducted the DSC, DSF, and SEC‐MALS analysis. PR, YM, HH, and SK conducted the CD analysis. SS and RW designed and executed the animal model. SS, RW, SK, and IJ performed post‐trial analysis. PB, YM, HH, and MW performed the endolysosomal degradation assay and PB conducted the mass spectrometric peptide sequencing and analysis. JR and ROH assisted with allergic patient recruitment and analysis. SK, SS, RW, and AL wrote the first draft. All authors contributed to manuscript editing and revision.

## Supporting information

Figure S1

Figure S2

Supplementary Material

Table S1
