# Supplementary figures and images for: Effect of structural stability on endolysosomal degradation and T‐cell reactivity of major shrimp allergen tropomyosin
Source: Allergy. 2020 Jun 18;75(11):2909–19. doi: 10.1111/all.14410 (PMC7687109; doi:10.1111/all.14410)

Supplementary figure S2:

Pen m 1                      pH 4.5

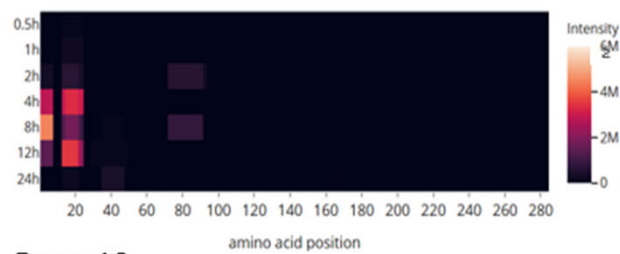

Bla g 7                      pH 4.5

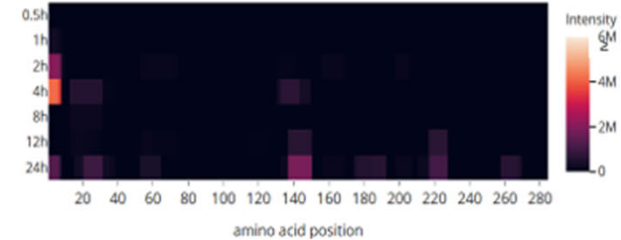

Der p 10                      pH 4.5

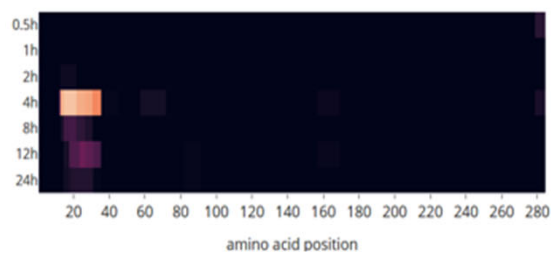

Ani s 3                      pH 4.5

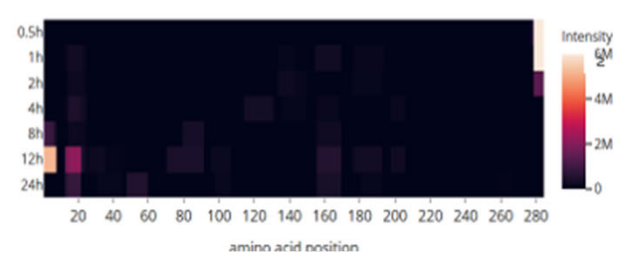

Supplement: Supplementary file 2 — Figure S2 [file ALL-75-2909-s002.pdf]
