# Supplementary material for: Effect of structural stability on endolysosomal degradation and T‐cell reactivity of major shrimp allergen tropomyosin
Source: Allergy. 2020 Jun 18;75(11):2909–19. doi: 10.1111/all.14410 (PMC7687109; doi:10.1111/all.14410)
Supplement: Supplementary file 4 — Table S1 [file ALL-75-2909-s004.docx]

**Supplementary Table S1:** Details on serum-based shrimp-specific and HDM-specific IgE levels and associated clinical symptoms for shellfish-allergic patients recruited for this study

| **Slot** | **IgE**  **(kU/L)** | **shrimp RAST (kUA/L)** | **HDM RAST (kUA/L) or SPT** | **Clinical symptoms** |
| --- | --- | --- | --- | --- |
| 1 | 199 | 10.10 | 8 mm (SPT) | Anaphylactic, eyes swelling, oral itch |
| 2 | 3401 | 6.65 | >100 | Urticaria, Angioedema |
| 3 | 127 | 1.65 | 0.09 | Lip swelling on contact |
| 4 | 242 | 1.32 | 11 mm (SPT) | Urticaria on handling |
| 5 | 288 | 9.82 | 2.66 | Facial oedema, red rash |
| 6 | 183 | 6.84 | 31.70 | NR |
| 7 | 579 | 3.63 | 5.03 | Oral/ hand contact |
| 8 | 266 | 17.2 | NT | Oral |
| 9 | 227 | 9.81 | 16.80 | Angiodema |
| 10 | 1946 | 9.50 | 14.10 | Oral |
| 11 | 323 | 21.60 | NT | Urticaria |
| 12 | 391 | 14.10 | 71.40 | Oral |
| 13 | 449 | 5.42 | 40.20 | Anaphylaxis, angioedema |
| 14 | 322 | 6.73 | 6.90 | Anaphylaxis, urticaria |
| 15 | 3424 | >100 | 22.00 | Angiodema, hives |
| 16 | 1665 | 12.6 | NT | Oral itch, exercise |
| 17 | 32 | 5.05 | NT | Itchy and tight throat |

NT – Not tested, NR – Not recorded, SPT – Skin prick test
